# Mapping of carbon monoxide related death risk in Turkey: a ten-year analysis based on news agency records

**DOI:** 10.1186/s12889-018-6342-4

**Published:** 2019-01-03

**Authors:** Günay Can, Uğurcan Sayılı, Özden Aksu Sayman, Ömer Faruk Kuyumcu, Duygu Yılmaz, Eren Esen, Eray Yurtseven, Ethem Erginöz

**Affiliations:** 1 Department of Public Health, Cerrahpasa Faculty of Medicine, Istanbul University-Cerrahpasa, Koca Mustafa Paşa Mahallesi, Cerrahpaşa Caddesi No:53, 34096 Fatih, Istanbul, Turkey; 2 Cerrahpasa Faculty of Medicine, Istanbul University-Cerrahpasa, Istanbul, Turkey

**Keywords:** Carbon monoxide, Carbon monoxide poisoning, Death risk, Unintentional poisoning, Turkey

## Abstract

**Background:**

Carbon-monoxide (CO) poisoning is a substantial cause of preventable mortality. In Turkey, no nationwide data are being collected nowadays. In our study, we aimed to assess the trend in deaths related to CO exposure in all provinces of Turkey in a 10-year period by using the records of a news agency which collects the news from the majority of the national newspapers, local newspapers and television channels.

**Methods:**

In this study, 27,881 news items that were released between January 2008 to December 2017 which included keywords of “poisoning” and “death” or “carbon monoxide” and “death” were evaluated. 2667 non-fire related deaths were used in the final analyses.

**Results:**

In a 10-year period, the risk of CO-related death in Turkey was 0.35/100000. 1371 (51.4%) of the victims were male and the median age of the patients was 45 years (range, 15 days-108 years). Most of the deaths occurred ≥50 years of age. Stoves were the predominant source [*n* = 2096 (78.6%)]. There was a stagnating trend of CO-related deaths. Most of the incidents occurred in winter. The Middle Anatolian region was of the highest risk in CO-related mortality.

**Conclusions:**

In conclusion, CO poisoning is still a considerable public health concern in Turkey. Results of our study showed that stoves are still frequently being used and are the cause of death especially in rural areas with lower socioeconomic status. A better organized, nationwide surveillance and management approaches are needed to demonstrate the true burden CO related morbidity and mortality as well as its prevention in Turkey.

## Background

Carbon monoxide (CO), which is termed as the “silent killer”, due to its tasteless and odorless nature, is an extremely lethal gas which is released from incomplete combustion of carbon-based fuels [1]. CO poisoning commonly results from indoor exposure to incompletely vented heating and boiling appliances, generators, and in some circumstances grills or car exhaust [2]. Higher affinity of CO to hemoglobin than oxygen leads to a hypoxic state which is the cause of the toxicity of CO poisoning [3].

CO poisoning is a substantial cause of preventable mortality. Although no universal data is available, CO poisoning is a known cause of more than a thousand deaths from 50,000 poisoning incidents in a developed country like US annually [4]. Additionally, irreversible neuropsychiatric sequela is a major concern after CO poisoning which increases its importance from a public health standpoint [5]. Besides the difficulties in detection of CO, the nonspecific flu-like symptoms of CO poisoning often lead to increased exposure, delayed hospital admission and delayed initiation of the treatment, which in turn increases both the morbidity and mortality related to poisoning [3].

The standardization in data collection regarding CO poisoning needs to be implemented worldwide [6]. In Turkey, no nationwide data are being collected nowadays. The current reports regarding CO poisoning related deaths are from either single institution emergency department admissions [7] or autopsy reports from local forensic science institutes [8] which can’t be generalized to whole the population. Lack of reporting may result in the underestimation of a preventable health issue. In our study, we aimed to assess the trend in deaths related to CO exposure in all provinces of Turkey in a 10-year period by using the records of a news agency which collects the news from majority of the national and local newspapers.

## Methods

**Fig. 1:**
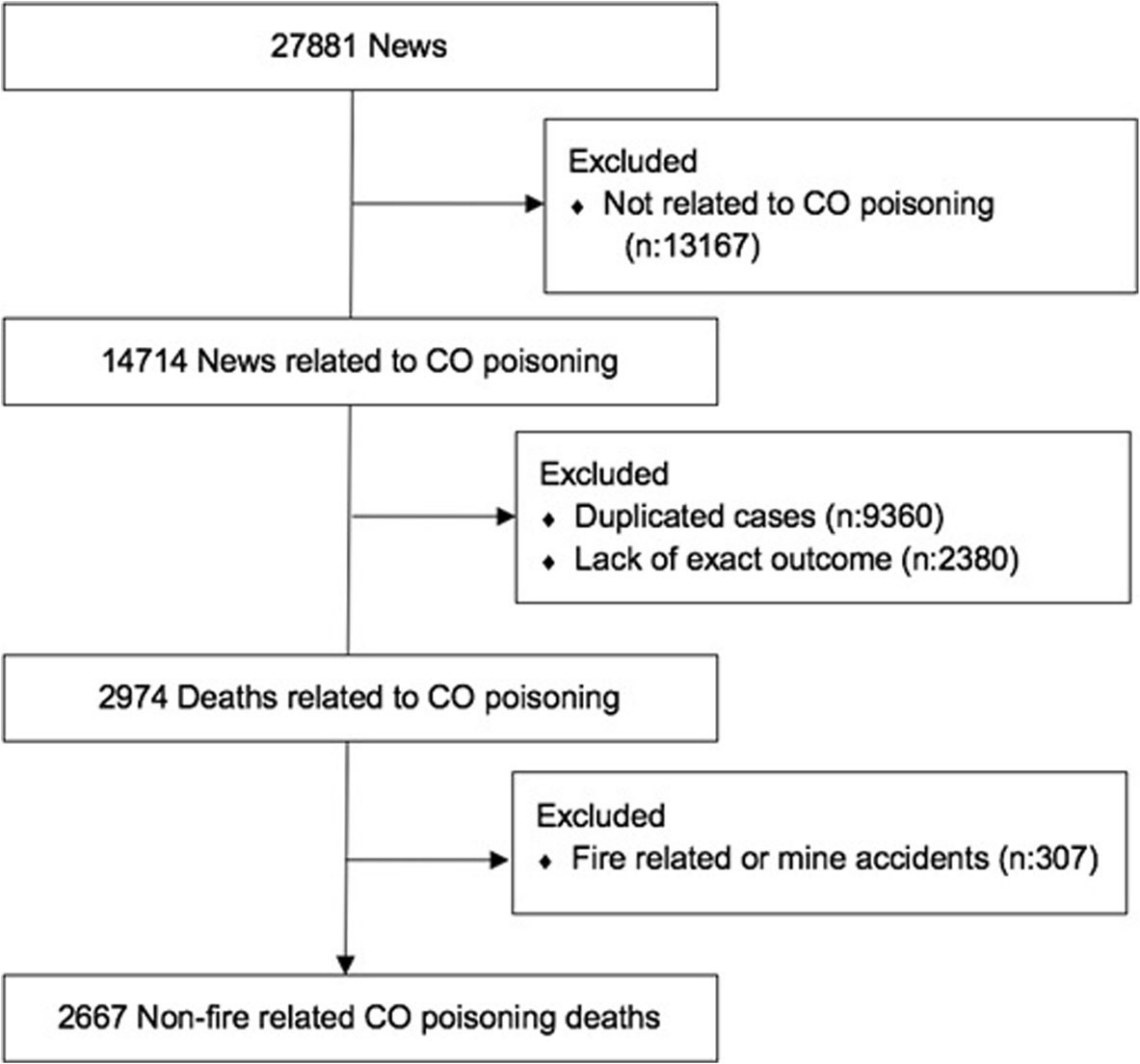
Flow diagram of the process to obtain study data

Similar to the methodology of Fisher et al. [9] the news items that were released between January 2008 to December 2017 which included the keywords of “poisoning” and “death” or “carbon monoxide” and “death” were obtained from the news agency. Media monitoring agency is a private company that collects many data from the press, television and radio-channels, websites, social media and other agencies [https://www.interpress.com/Detail/Page/Corporate]. In our analyses, 27,881 news items from 42 national newspapers, 488 local newspapers and 515 magazines were evaluated by 5 researchers. 14, 714 CO poisoning related news items were found. News items related to a CO poisoning incident were recorded by using the information of the victim’s name-surname, gender, age, the date of the incident, condition of the incident (work related, heating related, other), source of poisoning (stove, water heater-gas cylinder, gas heater, barbecue, machine related, other) and the province where the incident happened. Duplicate cases were removed by using the victims’ names. In most cases, the duplication in incidents which only included the initials of the victim’s or those which did not provide a name were determined by using the date of publication and the province. As a result, 5354 unduplicated incidents reamained. 2380 cases were excluded due to lack of exact outcome. Eventually, 2974 cases included CO related deaths. 307 of them were fire related or mine accidents and were excluded from analyses. Fire related deaths can not be precisely determined without forensic autopsy and mine accidents can be prevented with occupational health and safety protection methods rather than standard CO-protection methods. 2667 non-fire related deaths were used in the final analyses (Fig. 1).

**Fig. 2:**
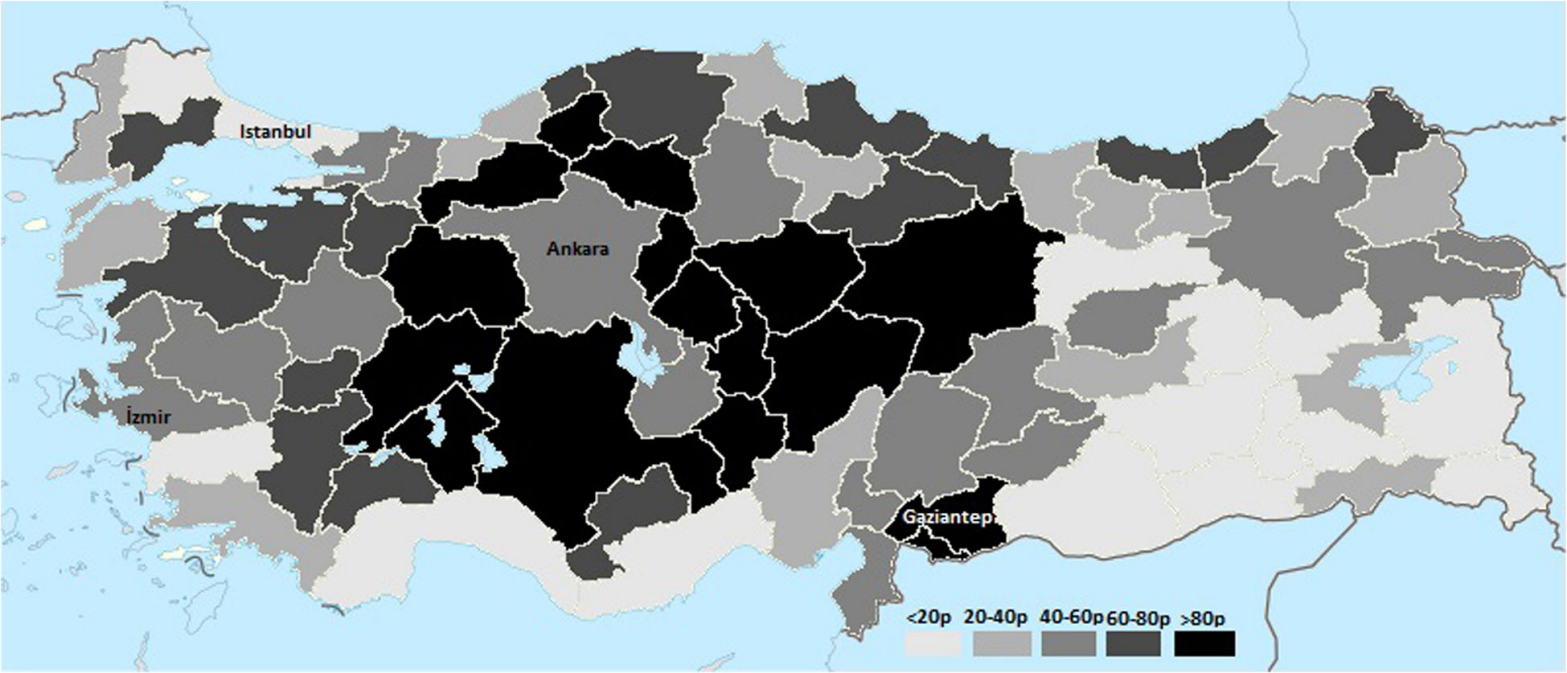
CO-related death risks in the provinces of Turkey (Colorless Turkey map template was provided from the Wikimedia Commons) [https://commons.wikimedia.org/wiki/T%C3%BCrkiye#/media/File:Turkey_location_map.svg]

The risk of CO-related death in the 10-year period of Turkey and its provinces was calculated by dividing the mean number of deaths in the 10-year period with the mean population of Turkey and its provinces in the same period. Population statistics were obtained from Turkey Statistical Institution data [10, 11]. The risk of CO-related death in particular provinces was divided into quintiles: the first quintile was accepted as having the lowest risk and the fifth quintile was accepted as having the highest risk. Considering the quintiles that the provinces belong to, a risk map was drew by using the colorless Turkey map as template. (Fig. 2) [12].

21st December – 20th March, 21st March – 20th June, 21st June – 22nd September, 23rd September – 20th December were accepted as winter, spring, summer, autumn respectively in order to show seasonal changes on CO poisoning.

Due to the use of publicly available data in this study, ethical approval is not needed. However, we obtained ethical approval from the Ethical Committee of the Cerrahpasa Faculty of Medicine (Date: 22.03.2018/Approval no: 34341879–604.01-02-109,722). The study was conducted according to the principles of the Declaration of Helsinki.

### Statistical analysis

The data were analyzed by using SPSS v20 and Microsoft Office Excel. Descriptive statistics were given as frequency (n) and percentage (%).

## Results

**Fig. 3:**
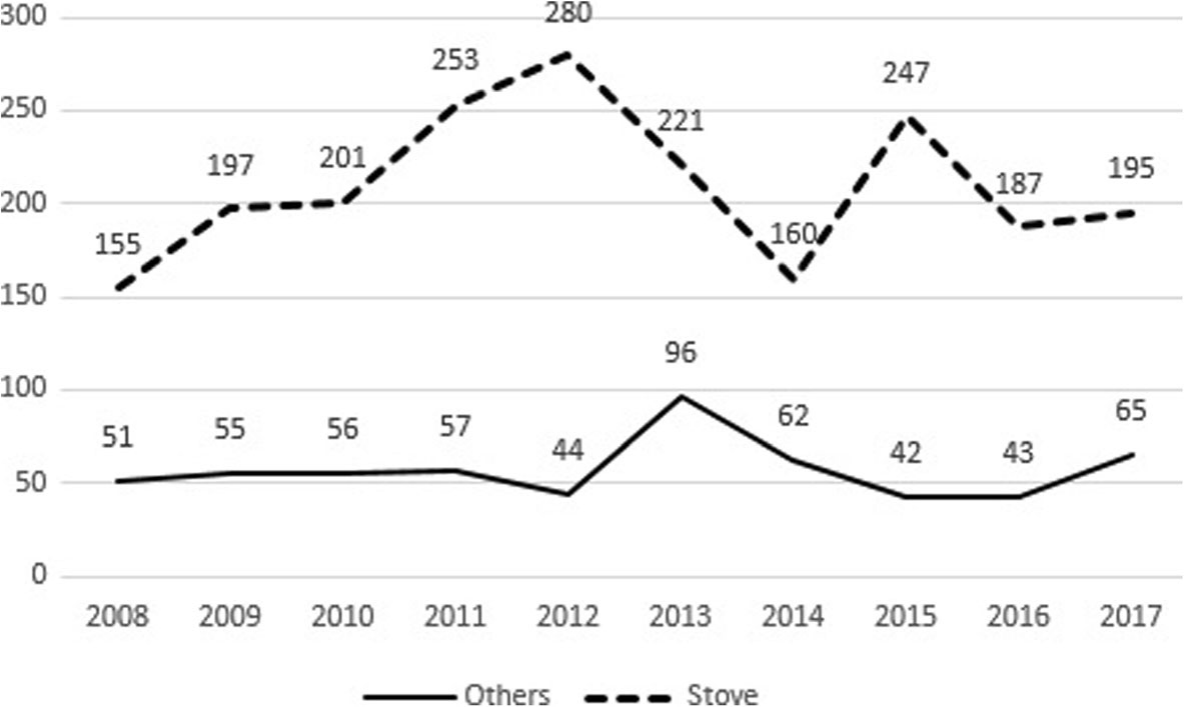
Trends in stove and non-stove related CO deaths in between 2008 and 2017

**Fig. 4:**
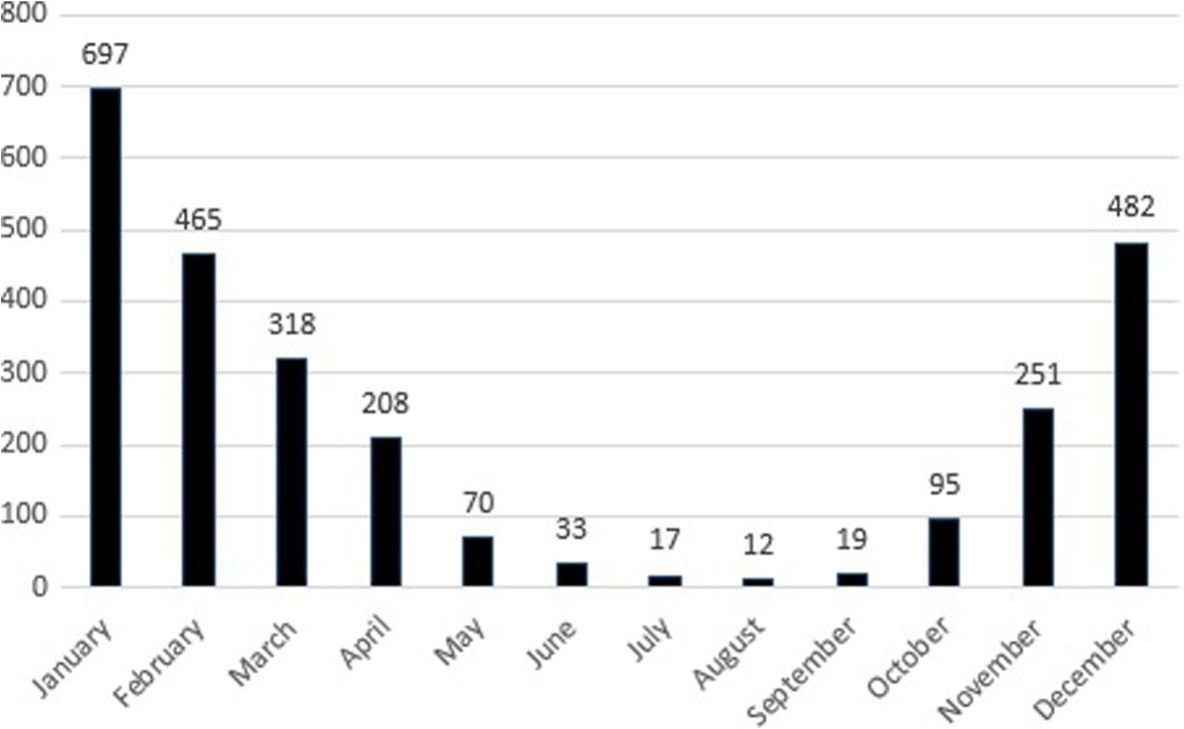
CO related deaths according to months

There were 2667 deaths from CO poisoning in the 10-year period. 1371 (51.4%) of the victims were male, 1178 (44.2%) of them were female and there were 118 (4.4%) victims whose genders were unknown. The median age of the patients was 45 years (range, 15 days-108 years). Most of the deaths occurred in the older age groups (≥50) and the distribution of the deaths in certain age groups is presented (Table 1). 2545 (95.4%) of the incidents were heating related, 50 (1.9%) of them were work related and 72 (2.7%) of them were unknown. Stoves were the predominant source [*n* = 2096 (78.6%)] and the number of the other sources that caused CO related deaths is shown (Table 1). Trends in stove and non-stove related deaths were depicted (Fig. 3). Most of the incidents occurred in winter (January) and the pattern of the deaths according to months is shown (Fig. 4).

**Table 1:** Sociodemographic characteristics of the CO related death victims

	Number	%
Gender		
Male	1371	51,4
Female	1178	44,2
Unknown	118	4,4
Age		
0–1	30	1,1
1–4	76	2,8
5–14	272	10,2
15–49	889	33,3
≥ 50	1095	41,1
Unknown	305	11,4
Condition of poisoning		
Work related	50	1,9
Heating related	2545	95,4
Other	72	2,7
Source		
Stove	2096	78,6
Water heater, gas cylinder	206	7,7
Gas heater	91	3,4
Barbecue	163	6,1
Machine related	32	1,2
Other	79	3,0

**Fig. 5:**
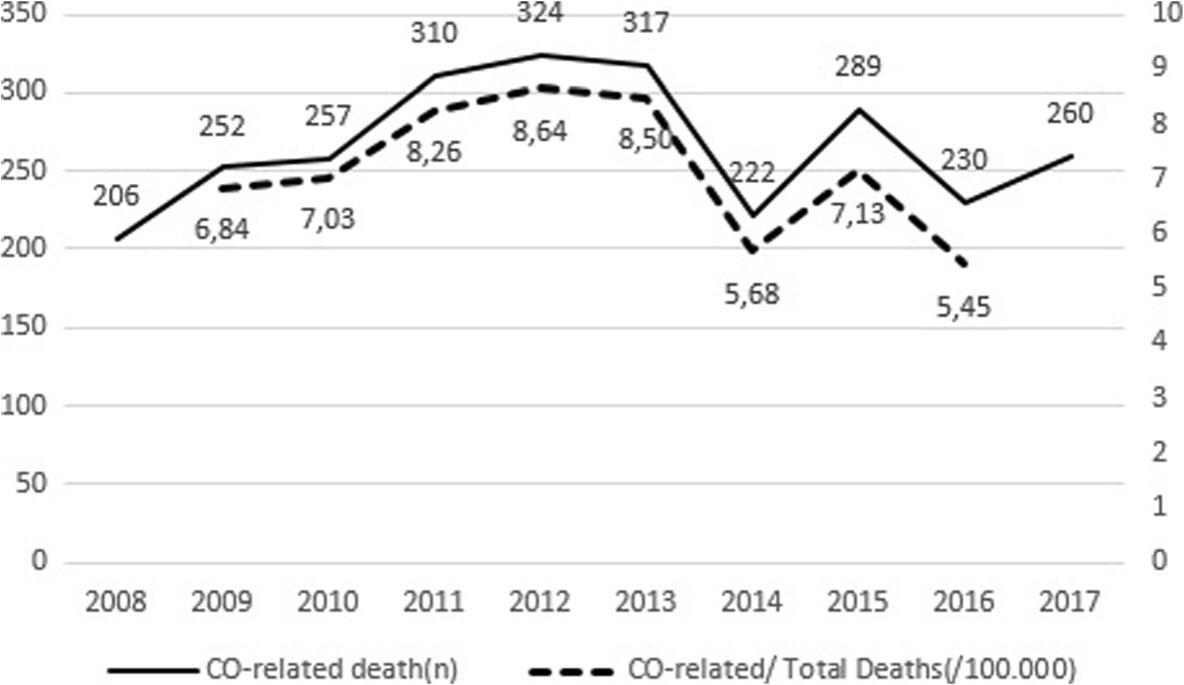
Trends in number of CO related deaths and the percentage in total deaths

There is a stagnating trend according to years and trends in CO related deaths and the percentage of deaths in total deaths are shown (Fig. 5).

The highest number of CO-related deaths was seen in Gaziantep (183 deaths in 10 year). The other provinces where deaths occured mostly are İstanbul (174), Ankara (158), Konya (154), Bursa (152). Higher numbers of deaths were seen in the provinces with relatively high populations.

In the study period, the risk of CO-related death in Turkey was 0.35/100000. The five provinces with the highest risk of death due to CO poisoning were Kilis (1.81/100000), Kırıkkale (1.78/100000), Karabük (1.31/100000), Niğde (1.28/100000), Kayseri (1.05/100000). 10 provinces with the highest death risk are shown (Table 2).

**Table 2:** Top 10 provinces with the highest risk of CO-related deaths in 10-year period

Province	Number of deaths	Death rate (/100,000)
Kilis	23	1.81
Kırıkkale	49	1.78
Karabük	30	1.31
Niğde	44	1.28
Kayseri	135	1.05
Gaziantep	183	1.01
Sivas	60	0.96
Bolu	27	0.95
Nevşehir	26	0.91
Yozgat	40	0.89
Turkey-Total	2667	0.35

## Discussion

Based on our results, it can clearly be seen that CO poisoning is a substantial public health problem in Turkey which kills hundreds of people annually. The stagnating pattern of CO related deaths in the 10-year period may show the underestimated importance of CO poisoning which may be caused by low death rates in previous reports. To our knowledge, there were only two nationwide reports showing 84 and 39 CO related deaths in the years 2008 and 2010 respectively, which does not seem realistic in a developing country [6, 13]. In our opinion, we may also have undercalculated the exact risk due to the fact that our methodology was only based on media reports. However, our study is the first report that shows the trend of CO related deaths in the last decade with more reasonable death rates in a developing country.

In a study conducted by the World Health Organization (WHO) in all its 53 Member States of European Region [6], despite heterogeneity in reporting between countries, the annual death rate of CO poisoning was found to be 2.24 per 100,000 population. In most countries, the trends of CO-related death rates were either steady or slowly decreasing similar to our findings. In that report, for instance, in Belarus and the Czech Republic the average annual death rates by CO poisoning were 11.99 and 2.62 per 100,000 population respectively. Considering the similar socioeconomic status of both of these developing countries to Turkey, more precise methods to show the real burden of the present health problem need to be applied. Local media in Turkey is a continuously working area that may have the potential to reach the farthest parts of Turkey. By evaluating their reports, we reached an average of 267 deaths per year that helps to emphasize the importance of the current problem to take preventive measures. Winter, unsurprisingly, was the season in which the death incidents peaked similar to other reports [14–16]. Also, we calculated the risk of CO-related death according to the provinces to discover the most vulnerable places to poisoning. The Middle Anatolian region had the highest risk of CO-related deaths. The Middle Anatolian region is a rural area with strong winters that could explain the widespread and relatively long utilization of stoves for heating purposes. Additionally, socioeconomic status is a known risk factor of CO poisoning [17]. The socioeconomic status in Turkey improves from east to west regions gradually. The socioeconomic status of Middle Anatolian region is relatively low compared to other parts of Turkey, other than the Eastern Anatolian region, which may have added to the increased risk of stove related deaths. Interestingly, in the Eastern Anatolian region, which has the lowest socioeconomic status in Turkey, relatively low CO-related death rates were observed. In our opinion, the lack of local media penetration into the Eastern parts of Turkey lead to the underreporting of CO poisoning related death incidents. Therefore, the risk of death may actually be higher in the Eastern Anatolian region than reported. Istanbul which is the most developed province in Turkey, have lower Co-related death rate. Major but relatively underdeveloped eastern province (Gaziantep) have 8 times more death rate compared to Istanbul. Ankara which is capital and second major developed city and izmir which is third major and developed city have similar death rate. But even here, there are twofold death rate compared to Istanbul. These differences can be thought to be due to the weather conditions and the density of natural gas use.

A study conducted by Metin et al., that investigated CO poisoning cases and related deaths in 2010 in Turkey using ICD-10 (International Statistical Classification of Diseases and Related Health Problems) coding system, showed only 39 CO related death incidents [13]. The majority of the poisoning cases, whose sources were known, were caused by stoves and show similar seasonal pattern with our study. However, in a developing country where stoves are still widely used, that number seemed extremely low and could indicate underreporting of CO poisoning incidents. We therefore evaluated media reports, where the incidents were recorded regularly, to obtain more logical findings. Certainly, underreporting still existed in our data, since incident reports from the Eastern Anatolia region, the coldest region of Turkey with a relatively low socioeconomic status, were extremely rare. However, 0.35 deaths per 100,000 population is the highest rate that has been reported so far. Another study executed by Akköse et al. from Bursa [7], evaluated CO related emergency department and intensive care unit admissions in 10 years’ period. All the poisoning cases were reported as accidents except one case which was reported as a suicide attempt. The majority of the poisoning cases were due to coal heaters which is in accordance with our study. Of the 305 admissions, 10 patients died in the study period. Considering Bursa is one of the provinces with high CO related death incidents in our study, those findings reveal that deaths could generally occur prior to hospital admission in CO poisoning. And also, local hospital admissions may show the real burden of the poisoning rather than single institution records. Other reports including CO-related deaths in Turkey are from medico-legal autopsies. In a review which was performed by Karapirli et al. [16], total 47,523 autopsies from 7 cities were evaluated within 27-year period. Although study period differed between provinces, 980 CO-related deaths were found. Although not report a comparable rate, the study is one of few studies and showing about 2% CO deaths of autopsies in Turkey. Taken into account significantly higher number of deaths from those reports, to obtain the nationwide burden of CO-related death incidents collaboration between forensic medicine, public health departments and emergency departments might be needed.

The results of our study showed that stoves are still frequently being used and are the cause of death especially in rural areas with lower socioeconomic status. The majority of the deaths occurred in domestic conditions with the use of old fashion stoves for heating purposes in accordance with the literature [18]. Currently, there are no proper measures being taken to prevent CO-related deaths in Turkey. This may have caused underestimation of the problem due to lack of data regarding to real burden of the poisoning. Certain measures can be taken to decrease the burden of the CO-related accidents. Increased use and regular maintenance of CO detectors have been shown to be an effective way to diminish the both morbidity and mortality related to the poisoning with additional cost saving benefits [19]. To decrease stove related poisoning incidents, encouragement to use natural gas in the both heating and cooking systems should be deliberated. If there is no alternative heating system without stoves, the use of quality coal should be encouraged. The content of coal (calorie, sulfur, etc.) and properties (humidity, ash etc.) affect the quality of coal and poor quality coal are reported to produce CO gas due to incomplete combustion [20]. Public education campaigns and prevention programs organized by the authorities’ can be reduced mortality and morbidity in winter months. Schools, governmental and non-governmental organizations can educate the community about the ventilation of places where coal stoves are used as heating system. Warnings on CO poisoning can be ensured through media and social media on risky days. Routine evaluation of the chimneys by professionals to prevent any blockage that may lead to backflow of the smoke is another crucial step to hinder CO to accumulate within closed places. Even though stove is the main source of poisoning, regular maintanence of water heater, gas heater and other engine should have done to prevent deaths. Although not common, using a motor vehicle, generator or any engine using gasoline within an improperly ventilated area should also be avoided [21].

### Limitations

There are several limitations of our study. First and foremost, we evaluated the death incidents from media reports without any forensic medicine-based diagnosis. By doing this, we may have included some deaths not related with CO poisoning. In addition, lack of media reports from some parts of Turkey might have failed to show the real risk in those areas. (to ignore this issue, not being able to make news due to more intensive agenda at that time, not worth publishing the death news which occurred after the incident, etc.) In our opinion, we may also have undercalculated the exact risk due to the fact that our methodology was only based on media reports. It can be possible that over-reporting due to population or interest in some regions. Also, there might be some duplications left due to misspelling or absence of victim names, despite this we tried to exclude the duplicated cases in many ways. However, this is a report which showed logical CO-related death rates in a developing country in a 10-year period.

## Conclusions

To conclude, CO poisoning is still a considerable public health concern in Turkey. A better organized, nationwide surveillance and management approaches are needed to demonstrate the true burden CO related morbidity and mortality as well as its prevention in Turkey.

## Abbreviations

CO: Carbon-monoxide
ICD-10: International Statistical Classification of Diseases and Related Health Problems
US: United States
WHO: World Health Organization
